# Implementing Screening and Brief Intervention in Community Pharmacies to Improve Medication Adherence

**DOI:** 10.1186/1940-0640-10-S2-P14

**Published:** 2015-09-24

**Authors:** Janice Pringle, Annette D Boyer, Mark H Conklin, Arnie Aldridge

**Affiliations:** 1 School of Pharmacy, Department of Pharmacy and Therapeutics, University of Pittsburgh, Pittsburgh, USA; 2 CECity®, Pittsburgh, USA; 3 Pharmacy Quality Solutions, Inc., Durham, USA; 4 RTI International, Research Triangle Park, USA

## Background

Improving medication adherence across the health care system is a vital component to improving patient outcomes and reducing downstream health care costs. Research suggests that pharmacists can be a highly effective tool for improving medication adherence when equipped with knowledge and skills on conducting adherence screenings and interventions.

The objective of this large scale pharmacy demonstration study is to evaluate the impact of a pharmacy-based intervention on adherence to five chronic medication classes.

## Material and methods

283 pharmacists from a national community pharmacy chain were assigned to the intervention group. Collectively, they screened 29,042 patients for poor adherence risk and provided brief interventions to individuals with an elevated risk. Results from these screenings and interventions were compared to those of a control group consisting of 295 pharmacists who screened 30,454 but did not provide any brief interventions.

## Results

Patients in the intervention group significantly improved adherence for all medication classes. Adherence for oral diabetes medications improved 4.8 percent. Adherence for beta-blockers improved 3.1 percent. Additionally there was a significant reduction in per patient annual health care spending for patients taking statins ($241) and oral diabetes medications ($341).

## Conclusions

This study demonstrated that interventions are a cost-effective tool that can be applied in health care sites across the country. Furthermore, pharmacies provide a yet untapped source of health care professionals who can perform screening and brief interventions with patients.

